# News

**DOI:** 10.1155/S1463924602000111

**Published:** 2002


					News
Reichhold appoints Regional Sales Director for
Central Europe
Research Triangle Park, NCo7 February 2002: Alex Car,
Vice-President of Sales for Reichhold' s Global Com-
posites, today announced the appointment of Ju
 rgen
Finke as Regional Sales Director for Central Europe.
Finke will be located in Aachen, Germany, and will be
responsible for all sales activities in Germany, Benelux,
Austria and Switzerland for Reichhold' s Global Com-
posites business. (Reichhold' s headquarters is based at
Research Triangle Park, NC, USA.)
Before joining Reichhold, Finke spent 25 years with
Owens Corning, where he played a key role in building
and developing the company' s European Composites
business. He held numerous sales, marketing and business
management positions, including Business Manager,
Sales Leader of Europe and Compression Molding
Marketing Manager.
Finke has speci(R) c commercial expertise in the com-
pression molding market and extensive contacts in the
German composites industry.
For more information on Reichhold, visit www.
reichhold.com
Journal of Automated Methods & Management in Chemistry
Vol. 24, No. 2 (March-April 2002) pp. 59
Journal of Automated Methods & Management in Chemistry ISSN 1463 9246 print/ISSN 1464 5068 online # 2002 Taylor & Francis Ltd
http://www.tandf.co.uk/journals
DOI: 10.1080/14639240210132372
59

				

